# Oral guanfacine treatment ameliorates the ADHD-like symptoms caused by developmental manganese exposure

**DOI:** 10.1016/j.pnpbp.2025.111517

**Published:** 2025-10-05

**Authors:** Ellie Fisher, Stephane A. Beaudin, Barbara J. Strupp, Donald R. Smith

**Affiliations:** a Department of Microbiology and Environmental Toxicology, University of California, Santa Cruz, CA, USA; b Division of Nutritional Sciences and Department of Psychology, Cornell University, Ithaca, NY, USA

**Keywords:** Manganese, Impulse control, Attention, Sensorimotor skills, Attention deficit hyperactivity disorder, Infant development, Environmental exposure

## Abstract

Epidemiological studies have linked developmental manganese (Mn) exposure to increased risk of ADHD and related symptoms in children and adolescents. Rodent model studies have 1) confirmed causality by demonstrating that developmental Mn exposure can cause lasting ADHD-like symptoms, 2) revealed that these symptoms (in Mn-exposed animals) are accompanied by a hypofunctioning catecholaminergic system in fronto-cortical-striatal brain areas, and 3) demonstrated that methylphenidate is efficacious in ameliorating these ADHD-like symptoms in Mn-exposed animals. However, stimulant medications such as methylphenidate do not lessen symptoms in 25–30 % of children and adolescents diagnosed with ADHD, indicating the need for alternative ADHD medications. Guanfacine, a specific noradrenergic α_2A_ receptor agonist, has proven to be an effective non-stimulant ADHD medication, although it is unknown whether this drug is effective in treating the ADHD-like symptoms produced by developmental Mn exposure. The present study was designed to test this hypothesis. Additionally, due to the pharmacological specificity of guanfacine, its use may provide mechanistic insight into the role of noradrenergic dysfunction as a contributor to the Mn-induced impairments. Male Long-Evans neonatal rats were orally dosed with vehicle or Mn (50 mg Mn/kg/d) from postnatal day 1–21, and orally treated with guanfacine (0, 0.1, or 0.3 mg/kg/d) during behavioral testing as adults. The results revealed that developmental Mn exposure produced lasting impairments in impulse control, attention, and sensorimotor function, and that oral guanfacine was efficacious in ameliorating the Mn-induced impairments in all three functional domains, although the treatment duration needed for efficacy varied by functional domain. In addition, in control (unexposed) animals, there was little or no effect of guanfacine on any functional domain. There was also little effect of the drug in the Mn-exposed animals under trial conditions where Mn deficits did not emerge. These findings 1) demonstrate the efficacy of oral guanfacine to ameliorate the lasting ADHD-like symptoms caused by developmental Mn exposure, and 2) provide additional support for the hypothesis that hypofunctioning of the noradrenergic system contributes to these lasting Mn deficits. Collectively, these findings suggest that individuals with environmentally-induced ADHD, such as that induced by developmental Mn exposure, may benefit from oral guanfacine treatment.

## 1. Introduction

Attention-deficit/hyperactivity disorder (ADHD) is the most common behavioral disorder among children and adolescents (Faraone et al., 2021). Epidemiological studies have reported that elevated exposure to manganese (Mn) is associated with increased risk of ADHD-like symptoms and ADHD diagnoses in children (Bhang et al., 2013; Bouchard et al., 2007; Broberg et al., 2019; Capelo et al., 2022; Hong et al., 2014; Oulhote et al., 2014; Schullehner et al., 2020). These studies have raised concerns that elevated environmental Mn exposure may contribute to increased risk of ADHD and related symptoms in children, but they are unable to establish causality.

Animal model studies from our group and others have established that developmental postnatal Mn exposure causes a constellation of behavioral impairments mimicking those seen in ADHD children, including inattention, impulsivity, arousal dysregulation, and sensorimotor dysfunction (Beaudin et al., 2024b; Beaudin et al., 2017a; Beaudin et al., 2017b; Beaudin et al., 2013; Howard et al., 2024). These ADHD-like impairments are accompanied by dysfunction of the catecholaminergic system in fronto-cortico-striatal brain areas, including reductions in stimulated release of dopamine and norepinephrine, altered protein expression levels of transporters for these neurotransmitters, and altered expression of dopamine D1 and D2 receptors (Conley et al., 2020; Kern and Smith, 2011; Kern et al., 2010; Lasley et al., 2020). The putative role of a hypofunctioning catecholaminergic system in producing these ADHD-like impairments following developmental Mn exposure is further evidenced by findings that these impairments are ameliorated by oral methylphenidate (Ritalin), a specific antagonist to the dopamine and norepinephrine reuptake transporters (DAT and NET) (Beaudin et al., 2015; Beaudin et al., 2017b).

While methylphenidate is the most widely prescribed ADHD medication, stimulant medications such as methylphenidate do not lessen symptoms in 25–30 % of cases, and ~ 6–15 % of ADHD children discontinue use of stimulant medications due to adverse side effects (Barkley, 1976; Childress et al., 2020; Greenhill, 1995; Swanson et al., 1991). An alternative therapy for ADHD patients who do not respond favorably to methylphenidate is the drug guanfacine, a selective α_2A_-adrenergic receptor agonist (Mechler et al., 2022). Guanfacine has been shown to be efficacious in improving the cognitive and behavioral symptoms in ADHD children (Biederman et al., 2008; Hunt et al., 1995; Sallee et al., 2009), effects which have been linked to activation of postsynaptic α_2A_-adrenergic receptors in the prefrontal cortex (Arnsten et al., 1988; Arnsten, 2020).

Notwithstanding the clinical efficacy of guanfacine in treating ADHD symptoms, it is unknown whether it is efficacious in ameliorating the ADHD–like impairments produced by developmental Mn exposure. We addressed this knowledge gap by assessing the efficacy of guanfacine treatment in our rodent model of childhood Mn exposure. Specifically, our goals were two-fold: 1) To determine the efficacy of oral guanfacine for ameliorating the lasting attentional, impulse control, and sensorimotor impairments caused by developmental Mn exposure, and 2) gain mechanistic insights into the role of noradrenergic dysfunction as a contributor to the Mn impairments, given the α_2A_ receptor specificity of guanfacine. Findings from the present study will inform the potential efficacy of guanfacine as a non-stimulant treatment for environmentally-induced ADHD, and shed light on the extent to which noradrenergic system dysfunction contributes to the ADHD–like deficits caused by developmental Mn exposure.

## 2. Methods

### 2.1. Animals

A total of 128 male Long-Evans rats were used for neurobehavioral evaluation, and an additional 52 male and female littermates were used for blood and brain Mn analysis. Animals were born from 35 nulliparous timed-pregnant rats (Charles River; gestational age 16 days), which were delivered to the UCSC facility in two separate shipments 1 week apart, resulting in an offspring age range of 10 days during behavioral testing as adults. Twelve to 24 h after parturition (designated postnatal day 1, birth = postnatal day 0), each litter was culled to eight pups, with five or six males and the remainder females. Only one male/litter was assigned to a particular treatment condition (see the Supplemental material, Subjects housing, feeding, and accreditation).

### 2.2. Study design

The study utilized a between-subject design where animals were randomized at birth into control or Mn-exposed groups (*n* = 54–56 animals/group), and following weaning and cessation of Mn exposure further randomized in three guanfacine treatment groups, yielding a 2 Mn × 3 guanfacine between-subject factorial design (*n* = 9–18 animals/group).

### 2.3. Manganese exposure

Manganese exposure was carried out as described in our prior studies (Beaudin et al., 2017a; Kern et al., 2010). Briefly, neonates were orally exposed to 0 or 50 mg Mn/kg/d in a vehicle solution (5 % *w*/*v* stevia in Milli-Q^™^ water) from postnatal day 1–21. For Mn dosing, a 202.2 mg Mn/mL stock solution was prepared by dissolving MnCl_2_ 4H_2_O with Milli-Q^™^ water; aliquots of the stock solution were diluted with a solution of the natural sweetener stevia for oral dosing of the neonates. Dosing solutions ranged from 0.020 to 0.101 mg Mn/μL over the course of pre-weaning exposure as the animals gained body weight. Manganese was delivered once daily into the mouth of each pup (~10–25 μL/dose) via a micropipette fitted with a flexible polyethylene pipet tip (Fisher Scientific, Santa Clara, CA, USA). This Mn exposure regime produces relative increases in Mn intake that approximates the increases reported in infants and young children exposed to Mn via drinking water, diet, or both (see Supplemental materials, Manganese exposure protocol and rationale).

### 2.4. Guanfacine treatment

Guanfacine was administered orally once daily over a 20-day period from ~postnatal day 150–181 at clinically-relevant doses of 0, 0.1, or 0.3 mg/kg/d; for comparison, oral guanfacine doses used clinically for the treatment of ADHD symptoms range from 1 to 4 mg daily for children, and up to 7 mg for adolescents, which equates to ~0.1 to 0.2 mg/kg/d, depending on dose and body weight (Newcorn et al., 2022; Vilus and Engelhard, 2025). Prior rodent studies with guanfacine typically administered the drug via i.p. or s.c. injection (Fredriksson et al., 2015; Hains et al., 2015; Nishitomi et al., 2018), although one prior study attempted oral dosing, albeit unsuccessfully (Freund et al., 2019). The poor success of the oral guanfacine dosing route in preclinical studies is likely due to the drug’s unpalatability to rodents. However, as guanfacine is typically administered orally in clinical settings, the use of nonoral dosing routes in preclinical studies limits their clinical relevance due to different pharmacokinetic profiles of the drug under oral versus injection dosing routes. For this reason, we developed a procedure for administering guanfacine orally that would be acceptable to the animals. These efforts yielded an effective oral dosing regimen, in which the rats were fed a potato chip (Ruffles cheddar and sour cream flavored) adulterated with the drug solution, as described in the Supplemental Materials. Most animals consistently consumed even the highest guanfacine dose within 1 min (i.e., 0.3 mg/kg/d, equivalent to 0.135 mg/dose for a 450 g rat).

For guanfacine dosing, a 3.2 mg guanfacine/mL Milli-Q^™^ stock solution was prepared by dissolving 50 mg of guanfacine hydrochloride (Sigma-Aldrich Inc., St-Louis, MO) in 15.6 mL of Milli-Q^™^ water. Potato chip vehicles were broken down into ~1.5 cm^2^ pieces and dosed with volumes of 5.0–50 μL of guanfacine stock solution, depending on treatment group and animal body weight. Rats were provided their potato chip adulterated with guanfacine or Milli-Q^™^ water vehicle ~20 min before the start of daily behavioral testing and observed by staff to consume the entire potato chip, which typically occurred within a few seconds.

### 2.5. Behavioral testing

All animals were tested in a series of 5-Choice Serial Reaction Time Tasks (5-CSRTT) to evaluate learning, attention, and impulse control, and in the Montoya staircase test to evaluate sensorimotor function. Sixteen identical automated 5-CSRTT testing chambers fitted with odor delivery systems were used (#MED-NP5L-OLF, Med Associates, Inc., St. Albans, VT), as described previously (Beaudin et al., 2017a). Behavioral training began in young adulthood (~postnatal day 70), with 1 week of food magazine and nose-poke training, followed by testing on two visual discrimination learning tasks with a fixed cue duration and no pre-cue delay, followed by a series of visual focused and selective attention tasks, as previously described (Beaudin et al., 2017a) (see Supplemental materials, Behavioral testing procedures).

#### 2.5.1. Focused and selective attention tasks

In the first focused attention task, both the delay before cue presentation and the duration of the visual cue varied pseudo-randomly across trials. The pre-cue delay varied between 0, 3, 4, and 5 s and the cue duration was either 0.5 or 0.7 s. Daily test sessions involved 150 trials, and the task was administered for 12 daily testing sessions starting on ~postnatal day 140. The animals were then tested on a second focused attention task, which included pre-cue delays of 3 or 4 s and a cue duration of 0.5 or 1 s; animals were tested in this task for 6 daily sessions (150 trials/session) while receiving, for the first time, daily oral guanfacine treatment. For the second focused attention task, testing and guanfacine treatment began on ~postnatal day 155.

The subsequent selective attention task with olfactory distractors was designed to evaluate the rats’ ability to maintain a behavioral or cognitive set in the face of distracting olfactory stimuli (Beaudin et al., 2017a). The selective attention task included the same pre-cue delays used in the second focused attention task and a fixed cue duration of 0.7 s. In addition, on one third of the trials in each daily testing session, an olfactory distractor was presented pseudo-randomly 1–3 s before presentation of the visual cue (See Supplemental Material, Behavioral testing procedures). Animals were assessed on the selective attention task for 14 days starting on ~postnatal day 160 while they continued to receive daily oral guanfacine treatment. The pre-cue delays selected for all focused and selective attention tasks were based on prior studies from our group showing that these delays were effective at revealing attention and impulse control deficits caused by developmental Mn exposure (Beaudin et al., 2017a); see Supplemental material, Behavioral testing procedures).

#### 2.5.2. Dependent measures for the focused and selective attention tasks

Recorded responses for all attention tasks included premature responses (responses made after trial onset but before visual cue presentation), correct responses (responses made to the illuminated port following visual cue presentation), incorrect response (responses made to a non-illuminated port following visual cue presentation), and omissions (failure to respond within the 10 s response interval following visual cue presentation). A %Accurate outcome was calculated as [#correct responses / (#correct + # incorrect responses) x 100], while the % Premature outcome was calculated as [#premature responses / (#correct + #incorrect + # premature + #omission responses) x 100]. Details about the calculation of response outcomes and other response latency measures are provided in the Supplemental material (*5-CSRTT dependent measures*).

#### 2.5.3. Montoya staircase apparatus and procedure

Eight Plexiglas staircase devices were used to assess forelimb sensorimotor reaching and grasping skills, as described previously (Beaudin et al., 2013). The Montoya staircase test procedure included habituation and training, beginning on ~postnatal day 140, and daily testing beginning on ~postnatal day 160 using a colored-coded food pellet procedure, as previously described (Beaudin et al., 2015; Beaudin et al., 2013). Forelimb sensorimotor function was measured step-by-step for the number of pellets taken, the number of pellets eaten, the % Success, and the number of pellets misplaced. Specifics about the calculation of these response outcomes are described in the Supplemental Material (*Staircase test dependent measures*).

### 2.6. Blood and brain Mn levels

Blood and brain samples were collected at three ages: At 1) postnatal day 24 (littermates of the behaviorally-tested animals), 2) postnatal day 66 (also littermates of the behaviorally-tested animals), and 3) the end of the study (~postnatal day 185; the behaviorally-tested animals). Animals were euthanized via CO_2_ asphyxiation and exsanguination, and whole blood was collected from the surgically-exposed heart and stored in EDTA Vacutainers at −20 °C until analyses. Whole brain was removed and the hindbrain collected for Mn analyses and stored at −80 °C (the remaining brain was sectioned and stored for other outcomes). Blood and brain Mn concentrations were determined by inductively coupled plasma - mass spectrometry with an analytical detection limit 0.01 ng/mL, as described previously (Beaudin et al., 2013).

### 2.7. Statistical methods

The behavioral data were modeled by way of structured covariance mixed models (SAS version 9.4 for Windows) and according to the factorial design of the study phase. Fixed treatment effects included Mn exposure (two levels corresponding to the two exposure treatment groups), guanfacine treatment (three levels corresponding to the three guanfacine dose groups), depending on the study phase. Furthermore, the model included the within-subject factors pre-cue delay, cue duration, testing session block (typically 3 days/session block), odor distraction condition, and/or staircase step level, depending on the task and outcome analyzed. In all models, the random effect was rat to account for correlations within observations from the same animal. The significance level was set at *p* ≤ 0.05, and *p*-values between 0.05 and 0.10 were considered trends and are reported if the pattern of findings aids in clarifying the nature of the Mn or guanfacine treatment effects. Significant main effects or interaction effects were followed by single degree of freedom contrasts to clarify the nature of the effects, using the Student’s *t*-test for pairwise comparisons of least squared means. Blood Mn data were analyzed using mixed model analysis of variance and Tukey’s post-hoc test for pairwise comparisons.

## 3. Results

Below, we focus on the behavioral outcome measures that most clearly reveal the Mn impairments and guanfacine efficacy. Specifically, for the 5-CSRTT tasks we focus on %Premature responses, reflecting impulse control, and %Accurate responses for attentional function. For the Montoya staircase task of sensorimotor function, we focus on the number of pellets taken, eaten, and %Success.

In overview, the study revealed several findings: 1) They confirm our prior findings showing that developmental Mn exposure causes lasting deficits in impulse control, attentional and sensorimotor function; 2) they demonstrate, for the first time, that a clinically-relevant dose of oral guanfacine is efficacious in ameliorating the Mn deficits, although efficacy depended upon the duration of guanfacine treatment; and 3) they provide evidence that noradrenergic system dysfunction plays an important role in the lasting Mn deficits. The findings leading to each of these conclusions are presented below.

### 3.1. Assessment of treatment group randomization leading to exclusion of the 0.1 mg/kg/day guanfacine dose

As noted above, the Mn and control group animals were each randomized into three guanfacine dose groups after weaning. Because treatment with the drug did not begin until after the animals finished the first focused attention task, we had the opportunity to assess whether the randomization was effective in creating three groups of comparable behavioral performance before guanfacine treatment began. The analysis of the first focused attention task revealed, unexpectedly, that the animals assigned to the 0.1 mg/kg/d guanfacine treatment condition differed in terms of premature responding and attentional accuracy from those assigned to the 0 and 0.3 mg/kg/d guanfacine dose conditions – prior to the initiation of guanfacine treatment (see Supplemental Fig. 1). A similar conclusion was reached on the basis of data from the Montoya staircase task, where a significant Mn effect (relative to controls) on total pellets eaten was seen for the animals assigned to the 0.1 guanfacine dose, but not for the groups assigned to the 0 (vehicle) or 0.3 mg/kg/d guanfacine dose (Supplemental Fig. 2).

These analyses also indicated that the 0.1 mg/kg/d guanfacine dose had no effect on any behavioral performance measure in either control or Mn-exposed animals, based on the fact that attentional, impulse control, and sensorimotor performance of animals assigned to the 0.1 mg/kg/d guanfacine dose group was comparable in the final stages of the first focused attention task and the Montoya staircase training phase (i.e., before guanfacine dosing began), when compared to the beginning stages of the second focused attention task and Montoya staircase testing phase after guanfacine treatment had begun (see Supplemental Figs. 1 and 2).

Based on these findings, we concluded that the randomization had not been effective; i.e., that animals assigned to the 0.1 mg/kg/d guanfacine dose group differed from those assigned to the 0 and 0.3 mg/kg/d guanfacine groups – prior to drug treatment. As a result, and to avoid reaching erroneous conclusions, we omitted the control and Mn animals assigned to the 0.1 mg/kg/d guanfacine group from the statistical analyses. Thus, all final models and subsequent results described below utilized a 2 Mn × 2 guanfacine design, and included only the animals assigned to the 0 and 0.3 mg/kg/d guanfacine dose groups.

### 3.2. Developmental Mn exposure leads to lasting impairment in impulse control, and guanfacine ameliorates this deficit

**Fig. 1.**
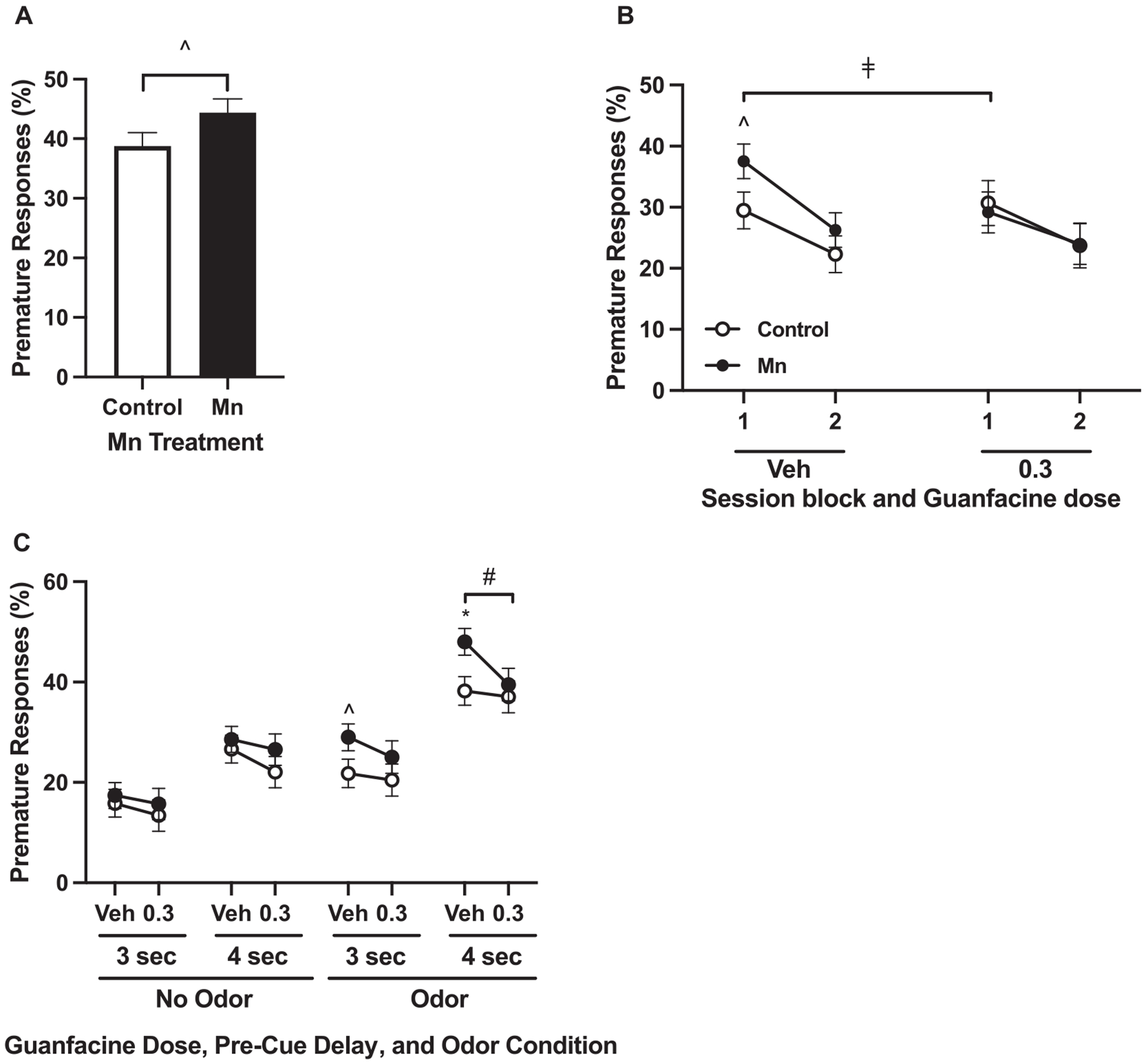
Developmental postnatal Mn exposure causes lasting deficits in impulse control and oral guanfacine ameliorates these deficits. (A) Mn exposure trends to increase %Premature responses compared to controls in the first focused attention task. (B) Mn exposure trends to increase %Premature responses in test session block one of the second focused attention task, and oral guanfacine treatment (0.3 mg/kg/d) alleviates this Mn deficit. %Premature responses for the control and Mn groups are shown as a function of test session block (3 daily test sessions/block). ǂ indicates Mn animals treated with guanfacine trend to commit fewer premature responses compared to their vehicle-treated counterparts in session block one (*p* = 0.062); ^*p* = 0.056 for Mn vs control in session block one. (C) In the selective attention task, Mn exposure increases premature responses compared to controls on trails with an odor distractor and pre-cue delays of 3 s (^*p* = 0.066) or 4 s (*p = 0.013), and oral guanfacine ameliorates this deficit. %Premature responses for the control and Mn groups, as a function of guanfacine dose, pre-cue delay, and odor distractor condition. # indicates Mn animals treated with guanfacine committed fewer premature responses compared to their vehicle-treated counterparts (*p* = 0.043). All data are least square means ± SEM (*n* = 51–52/Mn or control group in ‘A’, *n* = 9–17/group in ‘B’, and *n* = 11–16/group in ‘C’.

In the first focused attention task, there is a trending main effect of Mn exposure on %Premature responses, with the Mn-exposed animals committing more %Premature responses than controls [F(1, 65.9) = 3.10, *p* = 0.083]; no higher order interactions involving Mn were found (e.g., with pre-cue delay or test session block, *p* = 0.34, *p* = 0.48 respectively) (Fig. 1A).

**Fig. 2.**
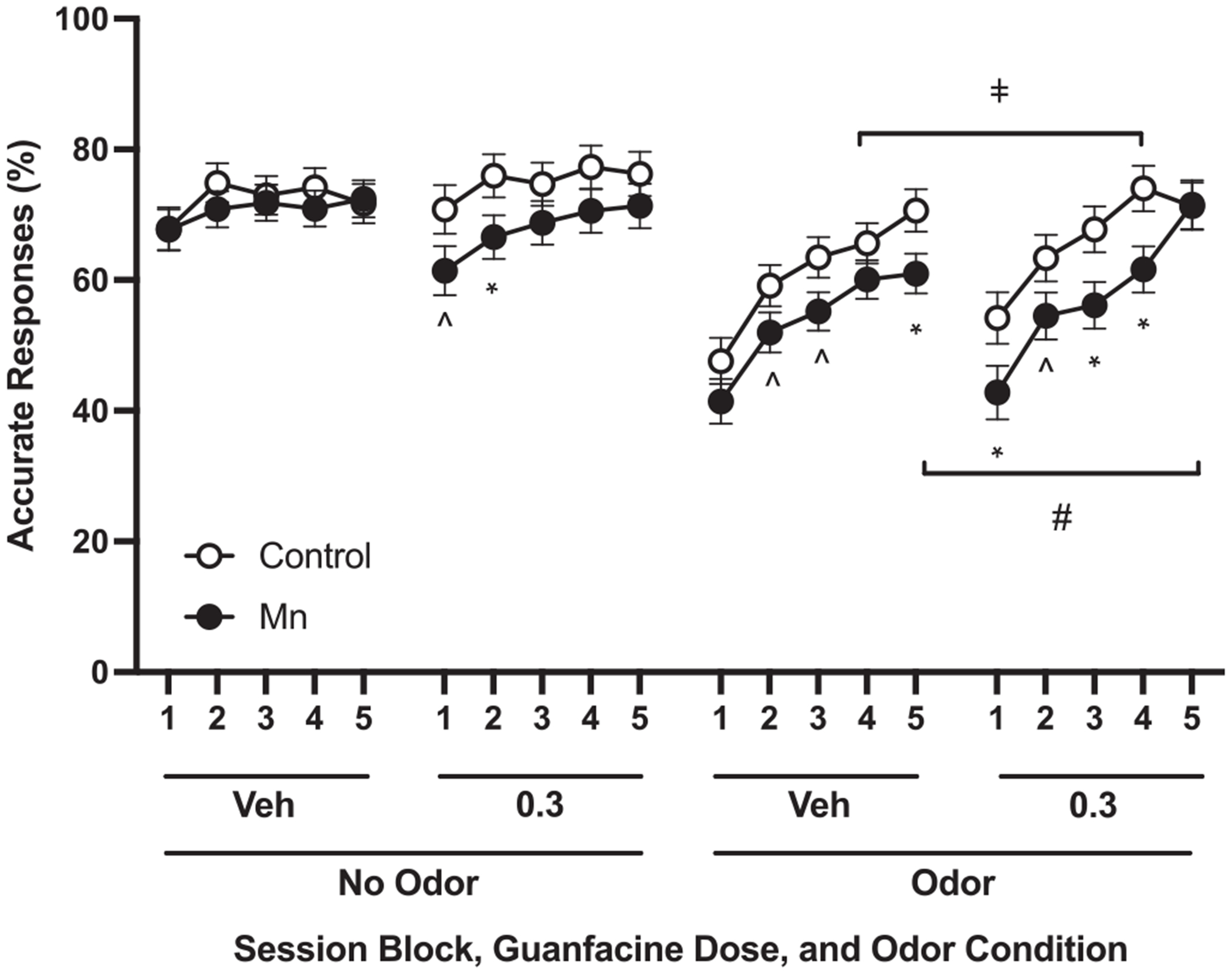
Prolonged oral guanfacine treatment ameliorates Mn-induced attentional deficits in the selective attention task with odor distractors. %Accurate responses for the control and Mn groups, as a function of test session block, guanfacine dose (0.3 mg/kg/d), and odor distractor condition in the selective attention task. Each session block contains 2–3 daily test sessions. Data are least square means ± SEM of the control and Mn groups (n = 11–16/group). *p ≤ 0.05 versus controls, ^*p* ≤ 0.1 versus controls, #*p* ≤ 0.03 versus 0 mg/kg/day guanfacine vehicle, and ǂp ≤ 0.07 versus 0 mg/kg/day guanfacine vehicle.

In the second focused attention task, during which oral guanfacine was administered, there is a trending Mn x guanfacine x session block interaction [F(1, 49.5) = 3.10, *p* = 0.084] for %Premature responses, reflecting that the effect of Mn on premature responses varies as a function of both guanfacine treatment and session block of testing (Fig. 1B). For the animals treated with vehicle (0 mg/kg/d guanfacine), a significant impulse control impairment due to developmental Mn exposure (vs controls) is seen in the first session block (*p* = 0.056), but this impairment does not persist into session block two, as impulse control in both control and Mn groups improve with increased testing. Further, the 0.3 mg/kg/d guanfacine dose completely ameliorates the Mn deficit in the first session block, based on two findings: 1) During this session block, %Premature responses by the Mn and control groups treated with guanfacine do not differ (Fig. 1B), and 2) the Mn group treated with guanfacine tended to exhibit fewer %Premature responses than their vehicle-treated counterparts (*p* = 0.062; Fig. 1B).

In the selective attention task, where odor distractors are presented pseudo-randomly before the visual cue on one-third of session trials, the analysis of %Premature responses reveals a significant four-way interaction of Mn exposure, guanfacine, pre-cue delay, and odor condition [F (1, 1167) = 4.00, *p* = 0.046] (Fig. 1C). This interaction reflects that the vehicle-treated Mn animals commit more premature responses than controls under odor distraction trials with a 3 s (trending *p* = 0.066) or 4 s (*p* = 0.013) pre-cue delay, and that oral guanfacine (0.3 mg/kg/d) completely ameliorates the increased impulsivity of the Mn animals (Fig. 1C). Specifically, the Mn animals treated with guanfacine committed fewer %Premature responses compared to their vehicle-treated counterparts on trials with a 4 s pre-cue delay (*p* = 0.043) (Fig. 1C). Notably, oral guanfacine has no measurable effect on %Premature responses in control animals in either the second focused attention or selective attention tasks (Fig. 1B, C).

### 3.3. Developmental Mn exposure causes lasting impairment in selective attention, which is ameliorated by prolonged oral guanfacine treatment

In the first and second focused attention tasks, there is no effect of developmental Mn exposure on attentional accuracy. Specifically, there is no main effect of Mn on %Accuracy in the first and second focused attention tasks (*p* = 0.747 and *p* = 0.291, respectively), and no higher order interactions involving Mn on %Accuracy in either task (p’s > 0.34 and p’s > 0.11). There are also no effects of guanfacine or higher order interactions involving guanfacine on attentional accuracy in either control or Mn groups in the second focused attention task (p’s > 0.11).

In contrast, in the selective attention task, again which included the presentation of odor distractors on a subset of trials, there is a significant Mn x guanfacine x odor x session block interaction for %Accurate responses [F(4, 1214) = 3.11, *p* = 0.015] (Fig. 2). This interaction reflects several influences: First, the introduction of the olfactory distractors significantly reduces attentional accuracy for both the control and the Mn animals, but to a greater extent in the Mn animals (e.g., in test session blocks two and three, p’s < 0.1 Mn vs controls, and in block five *p* = 0.031 Mn vs controls), reflecting a greater vulnerability of the Mn-exposed animals to the odor distractor (Fig. 2). Second, prolonged oral guanfacine treatment improves attentional accuracy in both control (session block four, *p* = 0.074 vs vehicle-treated controls) and Mn (session block five, *p* = 0.030 vs vehicle-treated Mn animals) groups. As a result, the significant deficit in selective attention in the vehicle-treated Mn animals in session block five is no longer seen in their guanfacine-treated counterparts (Fig. 2).

It is also notable that for selective attention task trials with no olfactory distractor, %Accurate responses in session blocks one and two are lower for the guanfacine-treated Mn animals than in their control counterparts treated with the drug, (i.e., *p* = 0.080 and *p* = 0.048, respectively), an effect that was not seen in the vehicle-treated groups (Fig. 2). This transient effect of guanfacine in the Mn animals, which is not present in session blocks three to five (i.e., with more prolonged testing and guanfacine dosing) may be attributed to the Mn animals treated with the 0.3 mg/kg/d guanfacine dose exhibiting a slightly slower rate of improvement in %Accuracy performance once guanfacine dosing started in the second focused attention task (see Supplemental Fig. 1B).

### 3.4. Developmental Mn exposure causes lasting deficits in sensorimotor function, and guanfacine ameliorates the Mn deficits

**Fig. 3.**
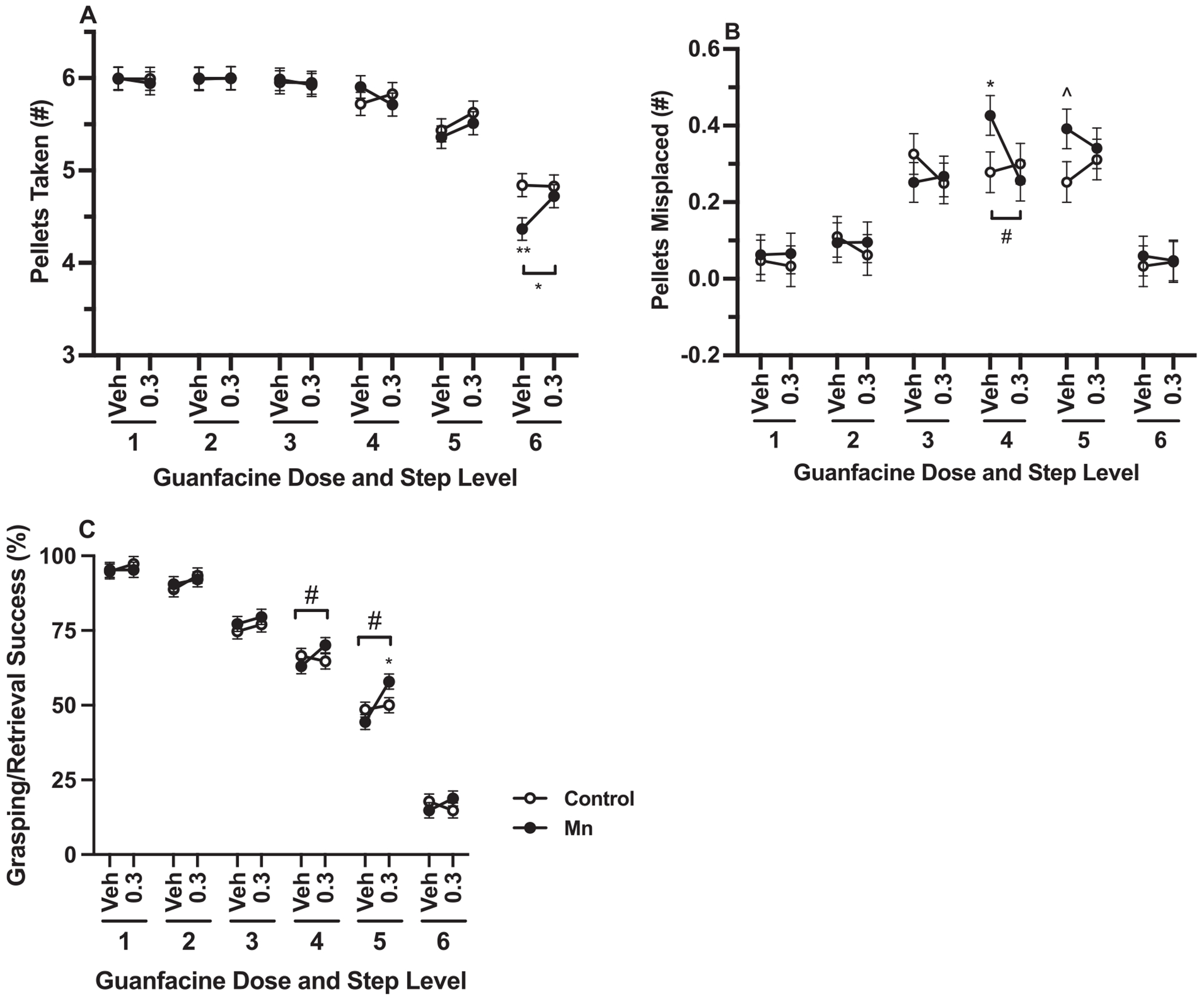
A–C. Developmental postnatal Mn exposure causes lasting sensorimotor deficits in the Montoya staircase test, and oral guanfacine treatment ameliorates these deficits. Data are (A) number of pellets taken, (B) number of pellets misplaced, and (C) percent grasping and retrieval success, as a function of guanfacine dose (vehicle or 0.3 mg/kg/d guanfacine) and staircase step. Data are least square means ± SEM of the control and Mn groups (n = 11–16/group). **p ≤ 0.007 versus controls, *p ≤ 0.05 versus controls, ^*p* ≤ 0.06 versus controls, and #p ≤ 0.05 versus 0 mg/kg/day guanfacine vehicle.

Developmental Mn exposure causes lasting impairment in sensorimotor function in the Montoya staircase test. Significant interactions of Mn x guanfacine x step were seen for analyses of the number of pellets taken [F(5, 6477) = 4.34, *p* = 0.0006], and the number of pellets misplaced [F(5, 6477) = 3.38, *p* = 0.0047], and oral guanfacine treatment fully ameliorated these Mn deficits (Fig. 3A, B). Specifically, the Mn group takes significantly fewer pellets than controls from the most difficult to reach step six (*p* = 0.0074) and misplaces significantly more pellets on steps four and five (p’s = 0.048 and 0.063, respectively), and oral guanfacine fully normalized these Mn deficits (Fig. 3A, B). As a result, Mn animals treated with 0.3 mg/kg/d guanfacine take significantly more pellets from step six and misplace significantly fewer pellets from step four compared to their vehicle-treated counterparts (p’s = 0.044 and 0.023, respectively) (Fig. 3A, B). Finally, there is also a significant Mn x guanfacine x step interaction for pellet grasping/retrieval %Success [F(5, 6477) = 8.49, *p* < 0.0001], which reflects that the Mn animals treated with guanfacine exhibited greater %Success on staircase steps four and five compared to their vehicle-treated counterparts (p’s = 0.048 and 0.0002, respectively) (Fig. 3C). Moreover, the Mn animals treated with guanfacine also show greater pellet grasping/retrieval % Success on step five compared to the guanfacine-treated controls (*P* = 0.028) (Fig. 3C). As with the impulse control (%Prematures) and attentional (%Accuracy) outcomes above, guanfacine treatment had no measurable effect on sensorimotor function in control animals (Fig. 3A–C).

### 3.5. Other behavioral outcomes

There *was no effect of Mn or guanfacine on omission errors or response latency on correct trials*. There is no main effect of Mn exposure or 0.3 mg/kg/d guanfacine treatment, or any higher order interactions involving Mn or guanfacine, on omitted responses (%Omissions) in either focused attention or selective attention tasks (data not shown). Further, there was no evidence that oral guanfacine treatment altered the correct response latency (i.e., latency for a nose poke in the correct port following presentation of the visual cue) for either the control or Mn groups in the second focused attention or selective attention tasks (p’s > 0.05 for guanfacine main effects or interactions involving guanfacine). Importantly, these findings indicate that the 0.3 mg/kg/d oral guanfacine dose did not produce any sedative effects in the control and Mn groups.

### 3.6. Developmental Mn exposure elevates blood and brain Mn levels, but levels are normalized by adulthood

Oral exposure to 50 mg Mn/kg/d over postnatal day 1–21 increased blood and brain Mn levels relative to their control counterparts, similar to our prior studies (Beaudin et al., 2017a; Beaudin et al., 2023; Kern et al., 2010). In postnatal day 24 littermates of the behaviorally tested animals, blood Mn levels in the Mn-treated animals (306 ng/mL ± 51.3 SE, *n* = 11) were significantly higher than controls (28.2 ng/mL ± 1.45 SE, *n* = 12) (*P* < 0.0001; Table 1). Similarly, brain Mn levels in postnatal day 24 Mn animals (11.0 μg/g ± 1.64 SE, n = 11) were significantly elevated compared to controls (3.39 μg/g ± 0.072 SE, n = 12) (*p* < 0.0001). By postnatal day 66, blood and brain Mn levels declined substantially, such that brain Mn levels were no longer elevated in Mn versus control animals, though blood Mn levels remained slightly elevated (Table 1). By the end of the study, blood Mn levels in the behaviorally tested animals (postnatal day 185) were not measurably different between control vs Mn groups (*p* = 0.83). For reference, mixed model ANOVA main effect statistics are: blood Mn, age F(2, 54) = 232, *p* < 0.0001, treatment F(1,54) = 81.2, *p* < 0.0001, age × treatment F (2,54) = 54.9, *p* < 0.0001; Brain Mn, age F(1,34) = 105, *p* < 0.0001, treatment F(1,34) = 41.1, *p* < 0.0001, age × treatment F(1,34) = 39.4, *p* < 0.0001). postnatal day, postnatal day.

## 4. Discussion

Epidemiological studies have linked developmental Mn exposures to increased risk of ADHD and related symptoms in children and adolescents (Bhang et al., 2013; Bouchard et al., 2007; Broberg et al., 2019; Capelo et al., 2022; Hong et al., 2014; Oulhote et al., 2014; Schullehner et al., 2020). Prior studies from our group have demonstrated causality, i.e., showing that developmental Mn exposure produces lasting ADHD-like symptoms in a rat model (Beaudin et al., 2015; Beaudin et al., 2024b; Beaudin et al., 2017b; Conley et al., 2020; Kern and Smith, 2011; Kern et al., 2010). In addition, studies using this animal model have revealed that these ADHD-like symptoms are accompanied by a hypofunctioning catecholaminergic system in fronto-cortical-striatal brain areas, and that methylphenidate, a DAT/NET antagonist, is efficacious in ameliorating the ADHD-like symptoms (Beaudin et al., 2015; Beaudin et al., 2017b; Beaudin et al., 2023; Conley et al., 2020; Kern and Smith, 2011; Kern et al., 2010). However, stimulant medications such as methylphenidate do not lessen symptoms in 25–30 % of ADHD children and adolescents, indicating the need for alternative ADHD medications. The present study addresses this need by further establishing that developmental Mn exposure causes lasting dysfunction in impulse control, attention, and sensorimotor function, and that a clinically-relevant dose of oral guanfacine treatment ameliorates the Mn-induced impairments. These findings and their significance are discussed below.

### 4.1. Impulse control is impaired by developmental postnatal Mn exposure, and guanfacine ameliorates this impairment

Behavioral inhibition is needed to perform well in 5-CSRT tasks with variable delays prior to cue presentation, as the animal must wait to respond until the light cue is presented (Bari et al., 2008; Cope et al., 2016). In the present study, developmental Mn exposure led to increased impulsiveness in adulthood, based on their increased incidence of premature responses in all three attention tasks (Fig. 1). All three tasks had unpredictable and variable delays prior to presentation of the visual cue, which challenges the animals to inhibit responding and wait for the cue. Additionally, in the selective attention task the presentation of olfactory distractors on a subset of trials served to markedly disinhibit both the control and Mn animals, but the effect was much more pronounced in the Mn-exposed animals (Fig. 1C). These findings are consistent with our prior studies (Beaudin et al., 2024b; Beaudin et al., 2017a; Beaudin et al., 2017b; Howard et al., 2024). They are also consistent with developmental Mn exposure increasing the risk of the predominantly hyperactive and/or combined hyperactive and inattentive ADHD phenotype, which in children and adolescents presents with a suite of symptoms or actions that include disruptive or impulsive behavior (Biederman et al., 2008; Cortese and Coghill, 2018; Faraone et al., 2021; Schullehner et al., 2020).

Notably, treatment with oral guanfacine, a specific noradrenergic α_2A_ receptor agonist, fully ameliorated the impairment in impulse control caused by developmental Mn exposure. This is evident in session block one of the second focused attention task where the Mn animals committed more premature responses than controls under the vehicle but not guanfacine treatment conditions (Fig. 1B). It is also evident in the selective attention task on trials with an odor distractor, where the Mn animals committed more premature responses than controls under the vehicle condition, but not when treated with guanfacine (Fig. 1C). In both tasks, guanfacine reduced premature responding in the Mn animals but had no effect on premature responding in controls. It is also note-worthy that the ability of guanfacine to reduce premature responding in the Mn-exposed animals cannot be attributed to an effect of the drug on sedation or motor function, given that the drug did not affect the latency to make a correct response in either the control of Mn-exposed animals.

Our finding that treatment with oral guanfacine improves impulse control in the Mn-exposed animals aligns with prior studies in which guanfacine treatment has been effective in clinical ADHD populations (Arnsten, 2020; Bédard et al., 2015; Biederman et al., 2008; Hunt et al., 1995; Muir and Perry, 2010; Sallee et al., 2009), and in animal models of ADHD (Fernando et al., 2012; Lukkes et al., 2020; Sagvolden, 2006). For example, double-blind clinical studies in 6–17 year old ADHD subjects found that guanfacine treatment was effective in decreasing hyperactivity/impulsivity symptoms (Biederman et al., 2008; Sallee et al., 2009). Further, using the SHR rat model of ADHD, Sagvolden reported that i.p. guanfacine doses of 0.3 and 0.6 mg/kg/d (but not lower doses of 0.075 and 0.15 mg/kg/d) reduced impulsiveness and overactivity in a visual discrimination task (Sagvolden, 2006). Similarly, in a study comparing rats with high and low levels of impulsivity in the 5-CSRTT (i. e., premature responding), Fernando and colleagues reported that systemic injection of 0.3 or 1 mg/kg/d guanfacine (but not lower doses of 0.03 and 0.1 mg/kg/d) decreased premature responses in the high impulsivity group, with no effect in the low impulsivity group (Fernando et al., 2012).

### 4.2. Attentional accuracy is impaired by developmental postnatal Mn exposure and guanfacine ameliorates this deficit

Symptoms of inattention, including increased distractibility, are features of both the inattentive and combined (inattentive plus hyperactive) phenotypes of ADHD (Drechsler et al., 2020; Leffa et al., 2022; Weibel et al., 2020). Here, developmental Mn exposure led to an impairment in selective attention in adulthood, as evidenced by the fact that they exhibited impaired attentional accuracy relative to controls on trials with olfactory distractors, an impairment not seen for the trials without distractors (Fig. 2). These findings are consistent with our prior studies (Beaudin et al., 2024b; Beaudin et al., 2017a; Beaudin et al., 2017b; Howard et al., 2024).

Oral guanfacine treatment, at a clinically-relevant dose (Newcorn et al., 2022; Vilus and Engelhard, 2025), significantly improved attentional accuracy in the Mn animals in the selective attention task, but only after prolonged dosing (i.e., after 18 days of drug treatment). Specifically, the efficacy of guanfacine is evidenced by the following patterns: 1) In session block five of the selective attention task, attentional accuracy was significantly lower for the vehicle-treated Mn animals than for their control counterparts, whereas the guanfacine-treated Mn and control animals performed identically, and 2) in this same session block five, attentional accuracy of the guanfacine-treated Mn animals was significantly better than their vehicle-treated counterparts (Fig. 2). Finally, there is also evidence of guanfacine improving attentional accuracy in the control group as well, based on a trending increase in accurate responses of guanfacine-treated controls in session block four of the selective attention task, compared to their vehicle-treated counterparts (Fig. 2).

Prior clinical and animal model studies have similarly shown that guanfacine treatment improves attentional function in ADHD and animal models of this disorder (Biederman et al., 2008; Hassani et al., 2024; Hunt et al., 1995; Sagvolden, 2006; Sallee et al., 2009). For example, double-blind studies of ADHD patients age 6–17 yrs. treated with oral guanfacine revealed that the guanfacine-treated children showed significant improvement on the inattentiveness subscales (Biederman et al., 2008; Sallee et al., 2009). Further, using the Connors questionnaire instrument, Hunt et al. found that oral guanfacine significantly improved mean scores overall in ADHD patients, including improvement in the Conners Inattention score (Hunt et al., 1995). Animal model studies have recapitulated these clinical findings. Using the SHR rat model of ADHD, Sagvolden found that guanfacine treatment ameliorated their impairment in sustained attention (vs the WKY rat strain) (Sagvolden, 2006). More recently, Hassani et al. demonstrated that guanfacine treatment (0.075 mg/kg i.m.) improved attentional function of nonhuman primates in an attentional set-shifting paradigm, as indicated by improved learning from errors and updating attention sets (Hassani et al., 2024). Moreover, these attentional benefits were accompanied by selectively enhanced neural signaling of prediction errors in neurons of the anterior cingulate cortex.

### 4.3. Sensorimotor function is impaired by postnatal Mn exposure and guanfacine ameliorates this deficit

ADHD is often co-morbid with psychomotor dysfunction (Bart et al., 2013; Brossard-Racine et al., 2012; Fliers et al., 2010; Kaiser et al., 2015; Pitcher et al., 2003; Watemberg et al., 2007), but it is unknown whether guanfacine is effective in lessening psychomotor impairments. To address this knowledge gap, we used the Montoya staircase test of sensorimotor function, which evaluates the animal’s ability to successfully take and eat small food pellets from the descending steps of the staircase; this includes advancing the limb over the food, opening the digits in preparation for grasping, grasping and manipulating the food pellet, and withdrawing the paw to place the food pellet in the mouth (Montoya et al., 1991). In the present study, developmental Mn exposure caused lasting sensorimotor impairments, with the Mn animals exhibiting 1) a significant decrease in the ability to take pellets from the lowest, farthest to reach step, and 2) a significant increase in the number of pellets that were misplaced from the fourth and fifth staircase steps, compared to their control counterparts (Fig. 3A and B). These findings are consistent with our prior studies of developmental Mn exposure (Beaudin et al., 2015; Beaudin et al., 2024a; Beaudin et al., 2013; Howard et al., 2024). In addition, we have shown in our prior studies that these sensorimotor impairments are alleviated by oral methylphenidate treatment (Beaudin et al., 2015; Beaudin et al., 2024a).

As noted above, little is known about the clinical efficacy of guanfacine to lessen psychomotor impairments associated with ADHD, and to our knowledge the present study is the first to test the effectiveness of oral guanfacine treatment to alleviate sensorimotor dysfunction in an animal model of ADHD. Here, we found that a clinically-relevant dose of oral guanfacine fully ameliorated the sensorimotor deficits caused by developmental Mn exposure. Specifically, we found that guanfacine ameliorated the Mn deficits in the rodents’ ability to reach and grasp pellets from the most difficult to reach step six, and the Mn deficits in the rodents’ ability to grasp pellets from steps four and five without dropping them (Fig. 3). Further, while there was no Mn deficit in the staircase outcome of %Success for grasping and retrieving pellets, the Mn group treated with guanfacine showed a significant increase in %Success at steps four and five, compared to their vehicle-treated counterparts (Fig. 3C). Altogether, developmental Mn exposure caused lasting sensorimotor impairments, and oral guanfacine ameliorated these Mn-induced sensorimotor impairments, as well as increased the success rate of pellet grasping/retrieval in the Mn-exposed animals.

### 4.4. Neural mechanisms underlying the therapeutic efficacy of guanfacine in Mn exposed animals

Reduced noradrenergic system function in the prefrontal cortex has been linked to ADHD symptoms in ADHD-diagnosed patients, possibly reflecting insufficient α_2A_-adrenergic receptor stimulation due to the low levels of norepinephrine (Smith et al., 2003). In animal models, reduced α_2A_-adrenergic receptor stimulation has been shown to produce impairments in locomotor activity, working memory, and impulse control (Ma et al., 2005). In previous studies, we have reported that developmental Mn exposure leads to lasting aberrations in catecholaminergic system function in the prefrontal cortex and striatum, including a decrease in protein expression levels of tyrosine hydroxylase, dopamine and norepinephrine transporters, and dopamine D1 receptors, an increase in the expression of D2 receptors, and a decrease in the evoked release of dopamine and norepinephrine (Conley et al., 2020; Kern and Smith, 2011; Kern et al., 2010; Lasley et al., 2020). Notably, however, our prior studies found no changes in α_2A_-adrenergic receptor levels in the prefrontal cortex due to developmental Mn exposure (Conley et al., 2020).

In the present study, the treatment duration of guanfacine efficacy differed for the impulse control versus attentional accuracy domains of Mn impairments, suggesting different mechanisms of efficacy. Specifically, the guanfacine-induced improvement in inhibitory control in the Mn animals was seen immediately (i.e., within the first session block of testing in the second focused attention task; Fig. 1B), whereas the benefits for attentional function were seen only after prolonged drug administration (i.e., after more than 18 days by the fifth session block of the selective attention task; Fig. 2). The immediate efficacy of guanfacine to ameliorate the impulse control deficits likely reflects the acute (i. e., pharmacologic) actions of guanfacine activating α_2A_-noradrenergic receptors and increasing neuronal firing. In contrast, the more prolonged time course seen for the benefit in attentional function suggests a mechanism that emerges only with repeated drug administration. One such possibility for the latter is activation of dorsal lateral prefrontal cortex postsynaptic α_2A_-noradrenergic receptors and the downstream modulation/inhibition of the cAMP-PKA pathway in postsynaptic neurons, with the overall result of increased synaptic firing and synaptic stabilization of prefrontal cortex connections (Arnsten et al., 1988; Arnsten, 2020).

## 5. Conclusions

There is compelling epidemiological evidence linking environmental Mn exposure to increased risk of ADHD and related symptoms (Bhang et al., 2013; Bouchard et al., 2007; Broberg et al., 2019; Capelo et al., 2022; Hong et al., 2014; Oulhote et al., 2014; Schullehner et al., 2020). Our rat model of developmental Mn exposure recapitulates a suite of ADHD-like symptoms (Beaudin et al., 2017a; Beaudin et al., 2017b; Beaudin et al., 2024b; Beaudin et al., 2013; Conley et al., 2020; Howard et al., 2024; Kern et al., 2010; Smith and Strupp, 2023), implicating a causal link between developmental Mn exposure and ADHD/ADHD-like symptoms in humans. Importantly the present study provides evidence, for the first time, that a clinically-relevant dose of oral guanfacine is effective in lessening the ADHD-like symptoms produced by developmental postnatal Mn exposure. Moreover, the effectiveness of the drug supports the hypothesis that hypofunctioning of noradrenergic systems in the prefrontal cortex may contribute to these impairments in Mn-exposed individuals. Finally, our findings provide support for the notion that patients with ADHD of environmental etiology may benefit from guanfacine treatment.

## Supplementary Material

1

## Figures and Tables

**Table 1:** Blood and brain Mn concentrations. Blood and brain Mn concentrations in littermates of the behaviorally tested animals [postnatal day (PND) 24, 66], and in the behaviorally tested animals at the end of the study.*

	PND 24		PND 66		End of Study (~PND 185)
	Blood Mn	Brain Mn	Blood Mn	Brain Mn	Blood Mn
Control	28.2 ± 1.45 (12)^A, a^	3.39 ± 0.072 (12)^A, a^	9.14 ± 0.51 (7)^A, b^	2.43 ± 0.15 (7)^A, b^	9.75 ± 1.92 (12)^A, a^
Mn	306 ± 51.3 (11)^B, a^	11.0 ± 1.64 (11)^B, a^	16.5 ± 1.88 (6)^B, b^	2.45 ± 0.08 (8)^A, b^	9.42 ± 1.78 (12)^A, c^

* Data are means ± standard error (*n*); blood and brain Mn levels in units of ng Mn/mL and μg Mn/g of dry tissue, respectively.

Upper-case A, B superscripts: Within an age group and tissue, treatment groups with different upper-case superscripts are statistically different from one another (*p* < 0.05).

Lower-case a, b, etc. superscripts: Within a treatment group and tissue, values across ages with different lower-case superscripts are statistically different from one another.

## Data Availability

Data will be made available on request.

## Appendix A. Supplementary data

Supplementary data to this article can be found online at https://doi.org/10.1016/j.pnpbp.2025.111517.
