# Supplementary material for: Oral guanfacine treatment ameliorates the ADHD-like symptoms caused by developmental manganese exposure
Source: Prog Neuropsychopharmacol Biol Psychiatry. Author manuscript; Available in PMC 2026 Jul 5. (PMC13333142; doi:10.1016/j.pnpbp.2025.111517)
Supplement: 1 [file NIHMS2184650-supplement-1.docx]

**Supplemental Materials**

**ORAL GUANFACINE TREATMENT AMELIORATES THE ADHD-LIKE SYMPTOMS CAUSED BY DEVELOPMENTAL MANGANESE EXPOSURE**

**Authors:** Ellie Fisher^1^, Stephane A. Beaudin^1^, Barbara J. Strupp^2^*, Donald R. Smith^1^*

^1^Department of Microbiology and Environmental Toxicology, University of California, Santa Cruz, CA, USA

^2^Division of Nutritional Sciences and Department of Psychology, Cornell University, Ithaca, NY, USA

*Co-corresponding authors

List of supplemental materials:

1. Oral guanfacine dosing method development and pilot study
2. Data cleaning process
   1. Supplemental Table 1.
3. Evidence supporting the conclusion of ineffective randomization into the three guanfacine dosage groups.
   1. Supplemental Figure 1A-B
   2. Supplemental Figure 2
4. **Oral guanfacine dosing method development and pilot study**

Prior rodent studies with guanfacine typically, if not exclusively, administered the drug via injection (i.p. of s.c.) (Fredriksson et al. 2015; Hains, Yabe, and Arnsten 2015; Nishitomi et al. 2018), though at least one prior study attempted, unsuccessfully, an oral dosing route (Freund et al. 2019). The poor success of oral guanfacine dosing routes in preclinical studies is likely due to the drug’s unpalatability to rodents. However, in clinical settings guanfacine is administered orally, and the use of non-oral dosing routes in preclinical studies may diminish the clinical relevance of those studies due to different pharmacokinetic profiles of the drug under oral vs injection dosing routes. To address this, we conducted an oral guanfacine dosing pilot study where we explored different dosing vehicles to deliver oral guanfacine to rats.

A 3.2 mg guanfacine/mL dosing solution was prepared by dissolving guanfacine hydrochloride (Sigma-Aldrich Inc., St-Louis, MO) in 5% (w/v) stevia in Milli-Q^TM^ water. Eight adult female Long-Evans rats were used in the dosing pilot study. The rats were weighed daily to calculate the guanfacine dosing volume to deposit on the food vehicle to achieve an oral guanfacine dose of 0.3 mg/kg. For test dosing, two co-housed rats were placed in a dosing cage, without bedding and separated by a transparent plastic divider. In order to assess which food vehicle flavor was most palatable to the rats when dosed with vehicle (5% stevia in Milli-Q water) or guanfacine, three measures were recorded for each rat: 1) The relative amount of the food vehicle consumed (e.g., none, ~25, 50, 75, or 100%), 2) the amount of time for the rat to consume the food vehicle, within the maximum allotted time of 5 minutes, and 3) the number of times the food vehicle was picked up and then released by the rat. All dosing staff were blinded to the vehicle or guanfacine dose on the food vehicle.

We first explored pieces of Nabisco Nilla wafers as the food vehicle, since we had used these in prior studies to successfully deliver oral methylphenidate to rats (Beaudin et al. 2015, 2017, 2024). We found that rats refused to consume the wafer when dosed with 0.3 mg/kg guanfacine. Similar negative results were obtained using other sugary cookie vehicles (e.g., Keebler Fudge Stripes Cookies). We surmised that the sugary cookie vehicles did not adequately mask the bitter taste of guanfacine. Subsequently, we explored a variety of salty potato chip vehicles to deliver the guanfacine, including Lays plain, BBQ, Sour Cream and Onion, and Ruffles Sour Cream & Cheddar flavors. Specifically, for the first 3 days of pilot testing, plain Lays potato chips were used, with the first day used to allow the rats to acclimate to the chip itself (i.e., without added guanfacine). While the rats showed improvement in how well they consumed the chip, only ~50% of the rats fully consumed the chip within the 5-minute timeframe by the third day of testing.

We then assessed testing with a variety of different flavored chips, including Lays BBQ, Sour Cream and Onion, and Ruffles Sour Cream & Cheddar. All three flavored chips were given to each of the rats at once and the order in which they consumed the chips was recorded. Overall, the Ruffles Sour Cream & Cheddar flavor was preferred (i.e., typically consumed first and fully consumed). The following day, the Lays plain and all three chip flavors were dosed with 0.3 mg/kg guanfacine, and again the Ruffles Sour Cream & Cheddar flavor was preferred. For the next 4 days, we dosed each pilot rat with guanfacine using only the Ruffles Sour Cream & Cheddar flavor potato chip and took all the three measurements noted above to assess consumption. By the last day of pilot testing, five out of the eight rats (62%) were consuming the whole guanfacine-adulterated chip within 1 minute, while two of the remaining rats (25%) consistently improved the rate at which they ate the chip, with only one rat not improving and eating only about a quarter of the guanfacine-adulterated chip. Based on these findings, we proceeded with oral guanfacine dosing using the Ruffles Sour Cream & Cheddar potato chip vehicle.

1. **Data cleaning process**

First, animals were removed from analysis for each task based on unacceptably low number of response trials that were performed, out of the maximum total of 150 response trials per test session (day). Specifically, animals completing less than response 75 trials per daily test session for more than 50% of test session days for a given task were removed from analysis for that task. Second, animals were removed from the second focused attention and selective attention task analysis based on poor or inconsistent guanfacine chip consumption. For this, we used the numerical scoring recorded for each animal’s guanfacine dose consumption for each day of dosing and testing. Animals were removed from the analysis for the second focused attention task and/or the selective attention task if they consumed less than half of the potato chip vehicle for more than 50% of treatment days in the task. Because the animals often improved their chip eating consumption with continued dosing, the selective attention task has more animals in the treatment groups then the second focused attention task. The final number of animals included in the statistical analyses for each behavioral task are shown in Supplemental Table 1.

**Supplemental Table 1**. The final number of animals per control, Mn, and guanfacine (0.3 mg/kg/d) treatment groups included in statistical analyses for each behavioral task. Animals were excluded from statistical analyses due to poor daily response trial performance or failure to consistently consume the guanfacine-dosed chip, etc.

| **Behavioral task** | **Control** | **Mn** | **Control + guanfacine** | **Mn + guanfacine** |
| --- | --- | --- | --- | --- |
| 1^st^ focused attention task (no guanfacine) | n = 52 | n = 51 | N / A | N / A |
| 2^nd^ focused attention task (guanfacine started) | n = 15 | n = 17 | n = 9 | n = 12 |
| Selective attention task (guanfacine con’t) | n = 14 | n = 16 | n = 11 | n = 11 |
| Staircase task (guanfacine con’t) | n = 14 | n = 16 | n = 11 | n = 11 |

1. **Evidence supporting the conclusion of ineffective randomization into the three guanfacine dosage groups.**

This study used a 2 Mn x 3 guanfacine between-subject experimental design, where control and Mn animals exposed over postnatal day 1-21 were randomized as young adults into one of three guanfacine treatment groups prior to initiating the oral guanfacine treatment phase of the study. In light of this between-subject design, we explored whether there was unexpected performance biases in animals randomized into the assigned guanfacine treatment groups before initiating guanfacine treatment, since any randomization bias might confound the interpretation of guanfacine efficacy. For this assessment, we generated 2 Mn x 3 guanfacine generalized linear mixed models for each of the three 5-CSRTT tasks (i.e., the first and second focused attention tasks and the selective attention task) and graphically displayed the groups’ performance for the outcomes of %Premature and %Accuracy (Supplemental Figure 1). A similar approach was followed for sensorimotor performance in the Montoya staircase task for the outcome of total pellets eaten (Supplemental Figure 2). These analyses revealed, unexpectedly, that control and Mn-exposed animals randomized to the 0.1 mg/kg/d guanfacine treatment group exhibited notably different performance in the first focused attention task (i.e., before guanfacine treatment actually started in the second focused attention task), compared to control and Mn-exposed animals randomized into the vehicle or 0.3 mg/kg/d guanfacine groups (Supplemental Figure 1). A similar performance bias was evident in this same group of animals in the Montoya staircase task (Supplemental Figure 2). While these analyses indicate that the 0.1 mg/kg/d guanfacine dose had no effect on any performance measure in either control or Mn-exposed animals, we chose to omit control and Mn groups assigned/treated with the 0.1 mg/kg/d guanfacine to avoid confounding influence in the statistical models. Therefore, all final models and subsequent results described in the main text utilized a 2 Mn x 2 guanfacine design, using the 0 and 0.3 mg/kg/d guanfacine dose groups.

**Supplemental Figures**


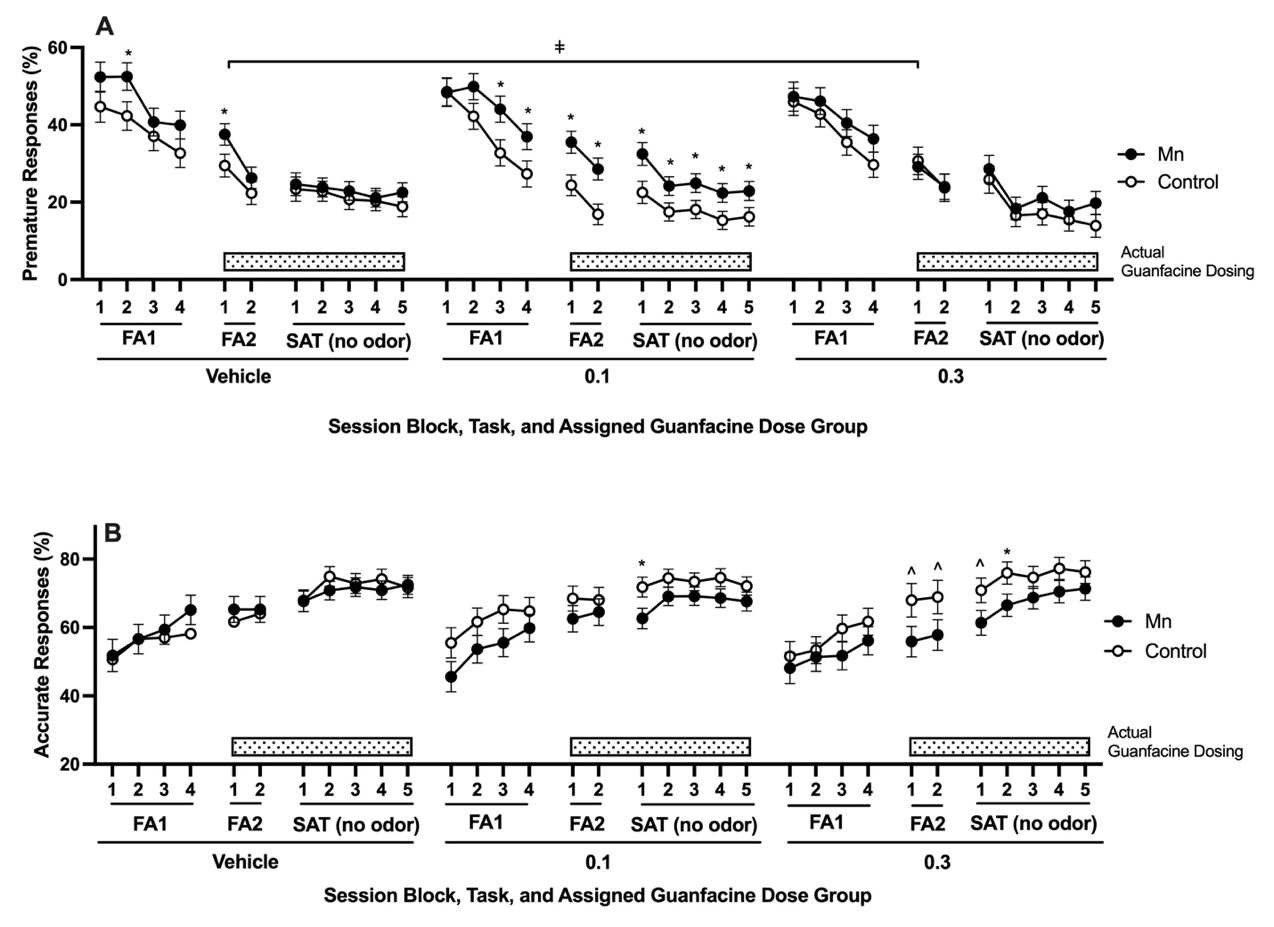


**Supplemental Figure 1A-B. Timeline of tasks split by assigned guanfacine dose group indicate ineffective randomization for the 0.1 mg/kg/d assigned group prior to starting guanfacine treatment.** The figure shows performance on the three separate tasks plotted on a common y-axis to reflect the timeline of behavioral outcomes for %Premature and %Accurate. Individual contrasts reflect contrasts obtained from the statistical models run on each separate task. The selective attention task (SAT) models included only the ‘No Odor’ trials. (A) Premature responses (%) for the control and the Mn groups, as a function of task session block, task, and assigned guanfacine dose group. (B) Accurate responses (%) for the control and the Mn groups, as a function of task session block, task, and assigned guanfacine dose group. Each test session block contains 2-3 daily test sessions. Data are least square means ± SEM of the control and Mn groups (n = 10 – 36/group). *p≤ 0.05 versus controls, ^p ≤ 0.1 versus controls, and ǂp ≤ 0.1 versus 0 mg/kg/day guanfacine vehicle.


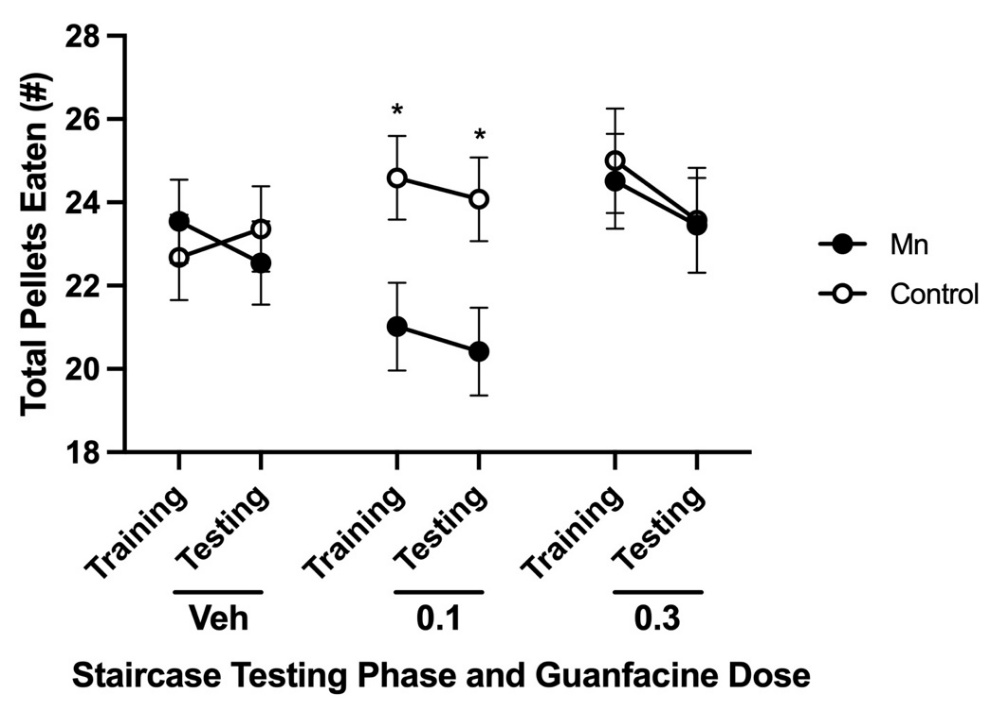


**Supplemental Figure 2. Performance on the training and testing phases of the Montoya staircase task of sensorimotor function by assigned guanfacine treatment group indicates ineffective randomization for the assigned 0.1 mg/kg/day guanfacine group prior to actually receiving guanfacine treatment.** Data are total number of pellets eaten by control and Mn groups across all steps as a function of staircase training/testing phase and assigned guanfacine dose group. Each training/testing phase contains three contiguous daily test sessions. Data are least square means ± SEM of the control and Mn groups (n = 11 – 16/group). *p≤ 0.05 versus controls.

**REFERENCES**

Beaudin, Stephane A., Shanna Howard, Barbara J. Strupp, and Donald R. Smith. 2024. “Methylphenidate Alleviates Cognitive Dysfunction Caused by Early Manganese Exposure: Role of Catecholaminergic Receptors.” *Progress in Neuro-Psychopharmacology & Biological Psychiatry* 131: 110949. doi:10.1016/j.pnpbp.2024.110949.

Beaudin, Stephane A., Barbara J. Strupp, Stephen M. Lasley, Casimir A. Fornal, Shyamal Mandal, and Donald R. Smith. 2015. “Oral Methylphenidate Alleviates the Fine Motor Dysfunction Caused by Chronic Postnatal Manganese Exposure in Adult Rats.” *Toxicological sciences : an official journal of the Society of Toxicology* 144(2). doi:10.1093/toxsci/kfv007.

Beaudin, Stephane A., Barbara J. Strupp, Walter Uribe, Lauren Ysais, Myla Strawderman, and Donald R. Smith. 2017. “Methylphenidate Alleviates Manganese-Induced Impulsivity but Not Distractibility.” *Neurotoxicology and Teratology* 61: 17–28. doi:10.1016/j.ntt.2017.03.005.

Fredriksson, Ida, Nitya Jayaram-Lindström, Malin Wirf, Erik Nylander, Erica Nyström, Kent Jardemark, and Pia Steensland. 2015. “Evaluation of Guanfacine as a Potential Medication for Alcohol Use Disorder in Long-Term Drinking Rats: Behavioral and Electrophysiological Findings.” *Neuropsychopharmacology: Official Publication of the American College of Neuropsychopharmacology* 40(5): 1130–40. doi:10.1038/npp.2014.294.

Freund, Nadja, Chloe J. Jordan, Jodi L. Lukkes, Kevin J. Norman, and Susan L. Andersen. 2019. “Juvenile Exposure to Methylphenidate and Guanfacine in Rats: Effects on Early Delay Discounting and Later Cocaine-Taking Behavior.” *Psychopharmacology* 236(2): 685–98. doi:10.1007/s00213-018-5096-0.

Hains, Avis Brennan, Yoko Yabe, and Amy FT Arnsten. 2015. “Chronic Stimulation of Alpha-2A-Adrenoceptors with Guanfacine Protects Rodent Prefrontal Cortex Dendritic Spines and Cognition from the Effects of Chronic Stress.” *Neurobiology of Stress* 2: 1. doi:10.1016/j.ynstr.2015.01.001.

Nishitomi, Kouhei, Koji Yano, Mika Kobayashi, Kohei Jino, Takuya Kano, Naotaka Horiguchi, Shunji Shinohara, and Minoru Hasegawa. 2018. “Systemic Administration of Guanfacine Improves Food-Motivated Impulsive Choice Behavior Primarily via Direct Stimulation of Postsynaptic α2A-Adrenergic Receptors in Rats.” *Behavioural Brain Research* 345: 21–29. doi:10.1016/j.bbr.2018.02.022.
